# GLP-1 and Dual GIP/GLP-1 Receptor Agonists in Pediatric Obesity: From Neuroendocrine Mechanisms to Clinical Application

**DOI:** 10.3390/ijms27188191

**Published:** 2026-09-15

**Authors:** Dominika Myśliwczyk, Małgorzata Myśliwiec, Alina Minarowska, Eliza Wasilewska

**Affiliations:** 1 Department of Pediatrics, Diabetology and Endocrinology, Medical University of Gdańsk, 80-210 Gdańsk, Poland; 2 Department of Pulmonology, School of Public Health, Collegium Medicum, University of Warmia and Mazury in Olsztyn, 10-719 Olsztyn, Poland; 3 Outpatient Cystic Fibrosis Clinic, Department of Pulmonology, Bialystok Medical University Children’s Hospital, 15-274 Białystok, Poland; 4 Division of Allergology, Medical University of Gdańsk, 80-214 Gdańsk, Poland

**Keywords:** pediatric obesity, GLP-1 receptor agonists, GIP/GLP-1 receptor agonists, tirzepatide, semaglutide, liraglutide, gut–brain axis, neuroendocrine regulation

## Abstract

Pediatric obesity is a chronic, multifactorial neuroendocrine disease in which disrupted gut–brain signaling, hypothalamic appetite regulation, adipose tissue dysfunction, and altered inter-organ communication sustain positive energy balance. Incretin-based therapies translate this biology into mechanism-based treatment, yet their developmental implications remain incompletely defined. This narrative review integrates evidence on GLP-1 and GIP receptor signaling with pediatric trial data and clinical implementation, with particular attention to developmental modifiers of gut–brain signaling and treatment during growth and puberty. We describe how GLP-1 receptor agonists act across central and peripheral tissues to reduce appetite, delay gastric emptying, and enhance glucose-dependent insulin secretion and examine the rationale for dual GIP/GLP-1 receptor agonism. Randomized pediatric trials demonstrate clinically meaningful BMI reduction with liraglutide and semaglutide, with the largest mean effect reported for semaglutide in adolescents; however, long-term effects on growth, puberty, bone health, body composition, and weight maintenance remain uncertain. Tirzepatide has shown greater weight-loss efficacy than selective GLP-1 receptor agonism in adults, but no randomized trial has evaluated it in pediatric obesity without diabetes. Incretin-based therapies should be integrated with nutritional, behavioral, psychological, and family-based care. Their future value will depend on durable efficacy, developmental safety, equitable access, and identification of patients most likely to benefit.

## 1. Introduction

Pediatric obesity has emerged as one of the major challenges facing modern medicine. It is now recognized as a chronic, relapsing, and multifactorial disease arising from complex interactions among genetic susceptibility, neuroendocrine regulation, metabolic dysfunction, environmental exposures, and behavioral factors. In children and adolescents, these mechanisms operate within the dynamic context of growth, pubertal development, and maturation of the central nervous system, adding further complexity to both disease progression and therapeutic response [1,2].

The neuroendocrine regulation of appetite and energy homeostasis plays a central role in the pathophysiology of obesity. The gut–brain axis integrates hormonal, neural, and metabolic signals originating from the gastrointestinal tract, adipose tissue, pancreas, and other peripheral organs within hypothalamic and brainstem circuits controlling hunger, satiety, reward-related eating, and energy balance. In pediatric obesity, impaired satiety signaling, leptin and insulin resistance, hypothalamic inflammation, and alterations in food-reward pathways may contribute to sustained excess energy intake and progressive metabolic dysfunction [3,4].

The incretin system has become an important therapeutic target within this neuroendocrine network. In addition to their established effects on glucose-dependent insulin secretion and glucagon regulation, glucagon-like peptide-1 (GLP-1) and glucose-dependent insulinotropic polypeptide (GIP) influence appetite, gastric emptying, nutrient handling, and inter-organ metabolic communication. Translation of this physiology into pharmacotherapy has led to the development of GLP-1 receptor agonists (GLP-1RAs) and, more recently, dual GIP/GLP-1 receptor agonists. By modulating several complementary pathways involved in appetite and metabolic regulation, these agents have expanded the therapeutic options available for obesity [5,6,7].

Clinical evidence supporting GLP-1RAs in children and adolescents is increasing, with liraglutide and semaglutide producing clinically meaningful reductions in body mass index and improvements in selected cardiometabolic outcomes. Dual GIP/GLP-1 receptor agonism represents a further therapeutic advance, although evidence in pediatric obesity remains limited. Important uncertainties persist regarding long-term safety, effects on growth and pubertal development, durability of treatment response, and optimal integration of pharmacotherapy into comprehensive obesity care. Moreover, mechanistic interpretations in pediatric patients continue to rely partly on evidence derived from adult populations and experimental models [2,7].

This narrative review examines the neuroendocrine mechanisms underlying incretin-based therapies in pediatric obesity. We integrate current knowledge of appetite regulation, gut–brain and inter-organ communication, and GLP-1 and GIP receptor signaling with evidence from pediatric clinical studies. We also discuss the clinical positioning of these therapies, unresolved developmental and safety considerations, and future directions for mechanism-based treatment of obesity in children and adolescents.

### Literature Search Approach

This narrative review was informed by a targeted, non-systematic search of PubMed for articles published between January 2020 and July 2026. Search terms included combinations of “GLP-1 receptor agonists”, “GIP/GLP-1 receptor agonists”, “tirzepatide”, “semaglutide”, “liraglutide”, “pediatric obesity”, “children”, “adolescents”, “gut–brain axis”, “appetite regulation”, and “mechanism of action”. Priority was given to randomized controlled trials, systematic reviews and meta-analyses, clinical guidelines, and mechanistic studies relevant to pediatric obesity and incretin receptor signaling. ClinicalTrials.gov and official regulatory sources, including the European Medicines Agency and the US Food and Drug Administration, were additionally searched to verify the status of ongoing pediatric trials and current therapeutic indications. Reference lists of selected publications were screened to identify additional relevant studies. Although emphasis was placed on literature published from January 2020 onward, earlier landmark studies were included when necessary to provide the physiological and mechanistic background.

Studies were considered for inclusion based on their relevance to the scope of the review, with particular emphasis on pediatric populations, clinical efficacy, safety, tolerability, mechanisms of action, and long-term treatment considerations. Randomized controlled trials and other high-quality clinical evidence were prioritized when comparing treatment efficacy and safety between agents. Adult studies were included when pediatric evidence was limited and when they provided relevant information on treatment mechanisms, discontinuation, long-term outcomes, or safety. Publications not directly relevant to the review objectives were excluded. The final selection of references was based on clinical relevance, methodological quality, and their contribution to the specific topics discussed in the review.

Throughout the review, statements supported directly by pediatric studies are distinguished from those extrapolated from adult trials or experimental models; where a mechanism is described based on animal or adult human data, this is stated explicitly and its pediatric relevance is identified as unverified.

## 2. Neuroendocrine Regulation of Appetite in Pediatric Obesity

The clinical effects of incretin-based therapies arise from the modulation of interconnected neural, endocrine, and metabolic pathways involved in appetite regulation and energy homeostasis. Although the fundamental organization of these pathways is broadly conserved across the lifespan, their activity is influenced by growth, pubertal maturation, and developmental changes within the central nervous system. Understanding this developmental context is therefore essential for interpreting the mechanisms and clinical effects of incretin-based therapies in children and adolescents.

### 2.1. Hypothalamic Regulation of Appetite

#### 2.1.1. Pathophysiology of Pediatric Obesity

The pathophysiology of pediatric obesity involves complex interactions between peripheral metabolic signals and central neural circuits regulating energy balance. The hypothalamus integrates information regarding nutrient availability and energy stores and coordinates responses affecting food intake, energy expenditure, and glucose metabolism. Within the hypothalamus, the arcuate nucleus (ARC) contains two functionally opposing neuronal populations that play a central role in appetite regulation [4,7].

Anorexigenic neurons express proopiomelanocortin (POMC) and cocaine- and amphetamine-regulated transcript (CART). Their activation promotes the release of α-melanocyte-stimulating hormone (α-MSH), which acts predominantly through melanocortin receptors MC3R and MC4R on downstream neurons to enhance satiety, suppress food intake, and promote energy expenditure. In contrast, orexigenic neurons expressing neuropeptide Y (NPY) and agouti-related peptide (AgRP) stimulate hunger and food intake while functionally antagonizing melanocortin signaling [4,5,6,7].

Obesity is associated with impaired communication between peripheral metabolic signals and these hypothalamic circuits. Elevated circulating leptin concentrations coexist with reduced central responsiveness to leptin, resulting in insufficient activation of anorexigenic POMC/CART pathways and impaired suppression of orexigenic NPY/AgRP signaling. Central insulin resistance may further weaken the anorexigenic effects of insulin and contribute to persistent dysregulation of appetite and energy balance [5,6,7,8].

Experimental evidence also indicates that chronic nutrient excess and obesity-associated low-grade inflammation can impair hypothalamic function through microglial activation and increased local signaling by pro-inflammatory mediators, including tumor necrosis factor-α (TNF-α) and interleukin-6 (IL-6). These processes may interfere with leptin and insulin signaling and promote the persistence of central energy-homeostasis disturbances. However, mechanistic evidence for hypothalamic inflammation is derived predominantly from experimental models, whereas direct evidence in children remains limited [5,7].

#### Pediatric Perspective

Unlike in adults, appetite regulation in children and adolescents operates within a continuously changing developmental environment. The hypothalamus retains considerable plasticity during growth and puberty, when neural circuits involved in energy homeostasis undergo remodeling alongside activation of the hypothalamic–pituitary–gonadal axis. Concurrent changes in sex steroids, growth hormone, insulin-like growth factor-1 (IGF-1), and insulin sensitivity influence appetite, substrate utilization, and energy requirements [9,10,11]. These developmental processes may modify the neuroendocrine response to pharmacological interventions. Consequently, the effects of GLP-1 receptor agonists in pediatric patients should not be interpreted solely through direct extrapolation from adult physiology. Nevertheless, evidence regarding age- and puberty-related differences in incretin receptor signaling and therapeutic response remains limited, representing an important area for future mechanistic and clinical research.

### 2.2. The Gut–Brain Axis

The gut–brain axis is a bidirectional communication network linking the gastrointestinal tract with the central nervous system. It contributes to the regulation of appetite, glucose homeostasis, gastrointestinal function, and energy balance through interconnected neural, endocrine, immune, and metabolic pathways. Signals generated in response to nutrient ingestion are transmitted through vagal afferent fibers, circulating hormones, microbial metabolites, and immune mediators and are integrated within brainstem and hypothalamic circuits controlling hunger and satiety [4,8].

Enteroendocrine cells represent a major sensory component of this network. In response to nutrients and other luminal stimuli, they secrete hormones including glucagon-like peptide-1 (GLP-1), glucose-dependent insulinotropic polypeptide (GIP), peptide YY (PYY), and cholecystokinin (CCK). Ghrelin, produced predominantly in the stomach, provides an opposing orexigenic signal. Together, these hormones coordinate meal initiation and termination, gastrointestinal motility, pancreatic secretion, and postprandial nutrient metabolism [8,12].

GLP-1 is produced by enteroendocrine L cells distributed throughout the intestine but enriched in the distal small intestine and colon. Following nutrient ingestion, endogenous GLP-1 enhances glucose-dependent insulin secretion, suppresses glucagon secretion through glucose-dependent and partly indirect mechanisms, and slows gastric emptying. Because circulating endogenous GLP-1 is rapidly degraded, its effects on appetite are mediated partly through local and vagal pathways involving the portal region and brainstem. In contrast, pharmacological GLP-1 receptor agonists have prolonged systemic exposure and can engage a broader range of central and peripheral GLP-1 receptor populations [4,8,13,14].

GIP is secreted predominantly by K cells in the duodenum and jejunum and contributes substantially to postprandial glucose-dependent insulin secretion. It also influences adipose tissue metabolism and energy storage, although its tissue-specific actions vary according to metabolic state and receptor context. Importantly, the effects of pharmacological GIP receptor activation in dual GIP/GLP-1 receptor agonists should not be considered equivalent to the physiological actions of endogenous GIP alone. Simultaneous pharmacological activation of GIPR and GLP-1R produces complementary effects on glucose regulation, appetite, and energy metabolism and may account for the greater weight reduction achieved with dual-receptor agonism [13,14].

Ghrelin is the principal circulating gut-derived hormone with a well-established orexigenic action. Its concentration typically rises before meals and decreases following nutrient ingestion. Ghrelin promotes hunger partly through activation of NPY/AgRP neurons in the arcuate nucleus. Conversely, PYY and CCK enhance satiation and satiety through vagal afferent signaling and effects on brainstem and hypothalamic circuits involved in the regulation of food intake [4,8].

Obesity is associated with impaired communication across the gut–brain axis, including reduced responsiveness to satiety signals, leptin and insulin resistance, altered vagal signaling, and changes in food-reward processing. These abnormalities may facilitate persistent excess energy intake and reinforce positive energy balance. Understanding these pathways has provided the physiological basis for incretin-based therapies, which modulate rather than fully restore the complex mechanisms governing appetite and metabolic homeostasis [4,7,12].

#### Pediatric Perspective

In children and adolescents, gut–brain communication operates within a developing neuroendocrine and metabolic environment. Although the intestinal microbiota acquires many adult-like characteristics during early childhood, its composition and functional capacity continue to be shaped by growth, diet, pubertal maturation, medication exposure, and environmental factors. Developmental changes may also occur in enteroendocrine signaling, vagal pathways, and central circuits regulating appetite, although their clinical relevance remains incompletely understood [15,16,17].

Microbial metabolites, particularly short-chain fatty acids, may influence energy homeostasis by activating free fatty acid receptors on enteroendocrine cells, modulating the secretion of GLP-1 and PYY, and affecting immune and neural signaling within the gut–brain axis [18]. However, direct evidence that developmental differences in the microbiota or gut–brain signaling modify the efficacy of GLP-1 receptor agonists in pediatric patients is currently limited. This relationship therefore remains a biologically plausible but insufficiently investigated area rather than an established determinant of treatment response [18].

### 2.3. Adipose Tissue as an Endocrine Organ

Adipose tissue functions not only as an energy reservoir but also as an active endocrine and immunometabolic organ. Adipocytes, preadipocytes, endothelial cells, fibroblasts, and resident immune cells within the stromal vascular fraction produce a broad range of hormones, cytokines, chemokines, and lipid mediators. These signals influence appetite regulation, insulin sensitivity, lipid metabolism, vascular function, and inflammatory responses in multiple organs, including the central nervous system, liver, skeletal muscle, and pancreas [6,8,19].

Among the best-characterized adipokines are leptin and adiponectin. Leptin is secreted primarily in proportion to adipose tissue mass and communicates the status of peripheral energy stores to hypothalamic circuits regulating food intake. Under physiological conditions, leptin suppresses appetite and supports energy homeostasis. In obesity, however, elevated circulating leptin concentrations coexist with reduced central responsiveness to the hormone. This state of leptin resistance limits activation of anorexigenic pathways and may contribute to persistent appetite dysregulation despite abundant energy stores [5,6].

Adiponectin generally exhibits an inverse relationship with adiposity. It enhances insulin sensitivity, promotes fatty acid oxidation, and exerts anti-inflammatory and anti-atherogenic effects. Reduced adiponectin concentrations in children and adolescents with obesity are associated with insulin resistance, dyslipidemia, and an unfavorable cardiometabolic risk profile. Consequently, the imbalance between elevated leptin and reduced adiponectin represents an important feature of adipose tissue dysfunction in pediatric obesity [8,19].

Expansion of adipose tissue in obesity is accompanied by cellular stress, altered adipokine secretion, and increased recruitment of immune cells. Rather than reflecting a simple transition between M1 and M2 macrophages, obesity-associated inflammation involves heterogeneous macrophage populations and complex interactions among innate and adaptive immune cells. Increased production of mediators such as tumor necrosis factor-α (TNF-α), interleukin-6 (IL-6), and monocyte chemoattractant protein-1 (MCP-1) promotes chronic low-grade inflammation and interferes with insulin signaling. These effects include inhibitory serine phosphorylation of insulin receptor substrate proteins and disruption of downstream insulin-signaling pathways, thereby contributing to systemic insulin resistance [19]. Adipose tissue inflammation and altered adipokine signaling may also influence hypothalamic function and gut–brain communication, further linking peripheral metabolic dysfunction with central appetite regulation. Incretin-based therapies can improve this adverse metabolic environment through weight reduction, decreased energy intake, improved insulin sensitivity, and attenuation of systemic inflammation. However, the extent to which GLP-1 receptor agonists act directly on human adipocytes remains uncertain, and many of their adipose tissue effects are likely indirect. In contrast, GIP receptor expression in adipose tissue is well established, although the metabolic consequences of GIPR activation depend on physiological conditions and the context of simultaneous GLP-1 receptor agonism [6,7,8,12].

#### Pediatric Perspective

Adipose tissue undergoes continuous expansion and remodeling throughout childhood and adolescence. Both adipocyte hypertrophy and hyperplasia contribute to this process, with their relative importance depending on developmental stage, duration of obesity, and individual biological susceptibility. Excessive hypertrophy promotes local hypoxia, cellular stress, immune-cell recruitment, and adipokine dysregulation, whereas the capacity to generate new adipocytes may partly determine whether excess lipid is stored within adipose tissue or deposited ectopically in the liver and skeletal muscle [20,21,22,23].

Early adipose tissue dysfunction may therefore produce metabolic consequences that persist into adulthood. At the same time, developmental differences in adipose tissue expandability, hormonal regulation, and inflammatory responses may influence the metabolic effects of incretin-based treatment. Direct evidence linking these developmental characteristics to differences in therapeutic response remains limited, and this relationship requires further investigation in pediatric populations [21,22,24].

### 2.4. GLP-1 and GIP Receptor Signaling

The glucagon-like peptide-1 receptor (GLP-1R) and glucose-dependent insulinotropic polypeptide receptor (GIPR) belong to the class B family of G protein-coupled receptors (GPCRs). Both receptors contain a large extracellular ligand-binding domain connected to a seven-transmembrane-domain structure responsible for signal transduction. Although GLP-1R and GIPR share several intracellular signaling pathways, differences in their tissue distribution, ligand–receptor kinetics, regulatory mechanisms, and cellular context contribute to distinct physiological and pharmacological effects [6,8,12,14].

GLP-1R and GIPR are highly expressed in pancreatic β-cells. Ligand binding predominantly activates the stimulatory G protein Gαs, leading to stimulation of adenylate cyclase and an increase in intracellular cyclic adenosine monophosphate (cAMP). The resulting activation of protein kinase A (PKA) and exchange protein directly activated by cAMP 2 (Epac2) facilitates membrane depolarization, calcium influx, mobilization of insulin-containing granules, and insulin exocytosis. These mechanisms amplify insulin secretion in a glucose-dependent manner, thereby limiting the risk of hypoglycemia when incretin receptor agonists are used without insulin or insulin secretagogues [7,8,12].

In addition to their acute effects on insulin secretion, GLP-1R and GIPR signaling can influence gene transcription, mitochondrial function, cellular stress responses, and β-cell survival. These longer-term effects involve cAMP-responsive transcription factors and interactions with phosphoinositide 3-kinase/protein kinase B and mitogen-activated protein kinase pathways. However, much of the evidence for direct β-cell proliferation and protection from apoptosis has been obtained from experimental models, and its relevance to human β-cell biology remains incompletely established [6,8,12].

The suppression of glucagon secretion by GLP-1 is mediated predominantly through glucose-dependent and indirect intra-islet mechanisms. GLP-1R activation in pancreatic δ-cells promotes somatostatin secretion, which inhibits α-cell activity and reduces glucagon release. Direct GLP-1R signaling in human α-cells remains debated because receptor expression appears low and varies among studies. Consequently, the reduction in hepatic glucose production observed during GLP-1R agonist therapy is likely to reflect a combination of reduced glucagon secretion, improved insulin action, delayed nutrient delivery, and indirect systemic effects [6,7,12].

Within the nervous system, GLP-1R is expressed in defined neuronal populations in the brainstem, hypothalamus, and other regions involved in appetite, autonomic regulation, and reward processing. Relevant structures include the nucleus tractus solitarius, area postrema, paraventricular nucleus, and selected neuronal populations within or connected to the arcuate nucleus. Pharmacological GLP-1R agonists reduce food intake through coordinated effects on central circuits and peripheral sensory pathways, including vagal afferent signaling. Their effects cannot therefore be attributed solely to direct activation of POMC/CART neurons or inhibition of NPY/AgRP neurons [7,12,14].

GLP-1R has also been identified in selected cell populations within the gastrointestinal, cardiovascular, and renal systems. However, interpretation of its extra-pancreatic distribution requires caution because receptor abundance is frequently low, expression varies between species, and some earlier localization studies relied on insufficiently specific antibodies. Moreover, the cardiovascular and renal benefits of GLP-1R agonists do not necessarily indicate direct receptor activation within every affected tissue. These outcomes may also result from weight reduction, improved glycemic control, altered hemodynamics, decreased inflammation, and interactions among multiple organs [6,7,12,14].

GIPR is abundantly expressed in pancreatic β-cells and is also present in selected neuronal populations, adipose tissue, bone, and the adrenal cortex. In β-cells, GIPR activates cAMP-dependent pathways similar to those initiated by GLP-1R and contributes substantially to postprandial insulin secretion. In type 2 diabetes, the insulinotropic response to physiological GIP is markedly reduced, although it may partially recover following improvement in glycemic control. Outside the pancreas, GIPR signaling influences adipose tissue metabolism, bone remodeling, and selected central nervous system pathways, although the physiological and pharmacological significance of these actions remains context-dependent [8,12,13,14,25,26].

#### Pediatric Perspective

The basic mechanisms of GLP-1R and GIPR signaling are likely preserved across different stages of life; however, data regarding developmental changes in receptor expression, receptor sensitivity, and tissue-specific responses in children and adolescents remain very limited. At the same time, growth and maturation involve substantial remodeling of the hypothalamus, hormonal axes, and adipose tissue, which may influence appetite regulation, energy balance, and metabolic responses [9]. Available clinical studies confirm the efficacy of GLP-1 receptor agonists in the pediatric population; however, they do not allow for a precise determination of the extent to which age and developmental stage modify receptor signaling and the therapeutic response [24,27,28]. For this reason, the biology of GLP-1 and GIP receptors in the pediatric population should not be considered merely as a direct equivalent of the mechanisms observed in adults.

**Figure 1:**
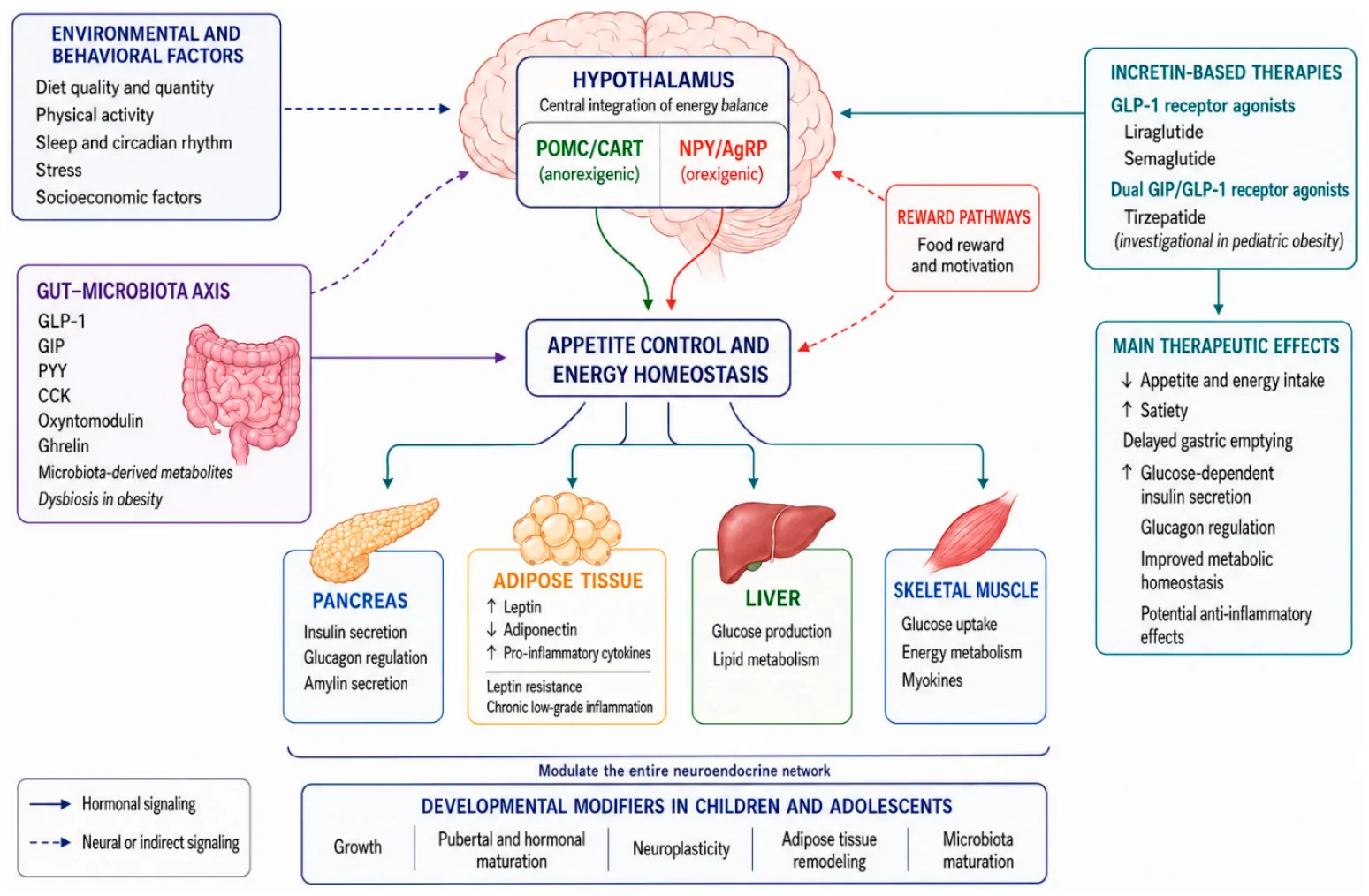
**Neuroendocrine regulation of appetite and therapeutic targets of incretin-based therapies in pediatric obesity.** The neuroendocrine regulation of appetite is coordinated by an integrated communication network linking the gastrointestinal tract, hypothalamus, pancreas, adipose tissue, liver, skeletal muscle, reward pathways, and gut microbiota. Peripheral hormonal signals, including GLP-1, GIP, peptide YY (PYY), cholecystokinin (CCK), ghrelin, and amylin, together with neural signaling via the vagus nerve, are integrated within hypothalamic appetite-regulating circuits (POMC/CART and NPY/AgRP neurons) to maintain energy homeostasis. In pediatric obesity, dysregulation of this network promotes leptin resistance, insulin resistance, chronic low-grade inflammation, and persistent positive energy balance. Developmental processes, including growth, puberty, neuroplasticity, adipose tissue remodeling, and maturation of the gut microbiota, may further influence these regulatory pathways and the response to pharmacological treatment. GLP-1 receptor agonists and dual GIP/GLP-1 receptor agonists act at multiple levels of this neuroendocrine network, modulating appetite and metabolic signaling, improving metabolic homeostasis, and ultimately contributing to clinically meaningful weight reduction and cardiometabolic improvement. Abbreviations: GLP-1, glucagon-like peptide-1; GIP, glucose-dependent insulinotropic polypeptide; PYY, peptide YY; CCK, cholecystokinin; POMC, proopiomelanocortin; CART, cocaine- and amphetamine-regulated transcript; NPY, neuropeptide Y; AgRP, agouti-related peptide.

The complex interplay between peripheral hormonal signals, hypothalamic appetite-regulating circuits and metabolic tissues, together with the developmental factors influencing these pathways in children and adolescents is illustrated in Figure 1.

### 2.5. Developmental Neurobiology of Arcuate Appetite Circuits: Why the Adolescent Circuit Is Not a Small Adult Circuit

The arcuate melanocortin system, involving POMC/CART and NPY/AgRP neurons, develops progressively and remains sensitive to metabolic and hormonal signals. Experimental studies indicate that leptin and early nutritional exposure can influence the organization of these circuits, highlighting the developmental dependence of appetite regulation [29,30]. In humans, although the initial formation of these pathways occurs predominantly prenatally and during early infancy, childhood and adolescence remain periods of functional maturation and neuroendocrine plasticity.

Puberty further modifies hypothalamic regulation through changes in gonadal steroids, leptin signaling, the kisspeptin–GnRH system, and the GH–IGF-1 axis [31]. Because energy availability influences both growth and reproductive function, sustained negative energy balance may potentially affect pubertal progression or growth. Whether the degree of energy restriction induced by GLP-1-based therapy is sufficient to produce such effects in adolescents with obesity remains unknown. This uncertainty supports systematic monitoring of growth and pubertal development in pediatric trials [32].

The long-term consequences of pharmacological modulation of appetite circuits during adolescence are also uncertain. GLP-1 receptor agonists act on hypothalamic, brainstem, vagal, and mesolimbic pathways involved in appetite, satiety, and food reward [33,34]. However, developmental data on GLP-1R and GIPR expression and signaling during human puberty are limited, and peripubertal preclinical studies remain scarce. This represents an important mechanistic evidence gap for the developmental use of incretin-based therapies.

#### The Affective and Psychosocial Interface of Feeding Circuits

Appetite-regulating pathways interact with neural systems involved in reward, motivation, and emotion. This may be particularly relevant during adolescence, when reward-related processes and prefrontal regulatory networks are still undergoing maturation [35]. Although GLP-1 receptor agonists may reduce food-related reward and improve weight-related quality of life, their effects on broader reward responsiveness, mood, body image, and eating behavior remain insufficiently studied [33,34].

Future pediatric trials should therefore assess not only metabolic and developmental outcomes but also relevant psychosocial measures, including mood, body image, eating behavior, and social functioning. Follow-up beyond active treatment will be important to determine whether potential benefits and behavioral effects persist after treatment discontinuation.

## 3. From Mechanisms to Therapy: Dual GIP/GLP-1 Agonism

### 3.1. Rationale for Dual GIP/GLP-1 Agonism

GLP-1 receptor agonists have substantially advanced the pharmacological treatment of obesity. Nevertheless, the magnitude of weight reduction varies among individuals, and a plateau commonly emerges during long-term treatment as compensatory mechanisms oppose further weight loss. These limitations have stimulated the development of unimolecular agents capable of engaging multiple, complementary pathways involved in appetite regulation and metabolic homeostasis [36,37].

Dual agonists targeting the glucose-dependent insulinotropic polypeptide receptor (GIPR) and glucagon-like peptide-1 receptor (GLP-1R) were designed to integrate the actions of two incretin pathways within a single molecule. This approach is intended to provide broader metabolic effects than selective activation of GLP-1R while maintaining coordinated pharmacokinetics and receptor engagement [13,25,38].

GLP-1 and GIP exert complementary but non-identical physiological actions. GLP-1 contributes to glucose-dependent insulin secretion, suppression of glucagon secretion, delayed gastric emptying, and reduced energy intake. GIP is a major postprandial insulinotropic hormone and also influences adipose tissue function, nutrient partitioning, and, according to experimental evidence, central pathways involved in energy balance. However, the effects of pharmacological GIPR activation in a dual agonist cannot be inferred directly from the physiological actions of endogenous GIP [13,25,38].

The role of GIPR signaling in obesity has long appeared paradoxical. Because endogenous GIP can facilitate lipid storage during nutrient excess, GIPR antagonism was initially proposed as an anti-obesity strategy. Conversely, sustained pharmacological GIPR agonism, particularly when combined with GLP-1R activation, also produces weight reduction and metabolic improvement in experimental models. This apparent contradiction indicates that the metabolic consequences of GIPR signaling depend on the duration and intensity of receptor activation, metabolic state, tissue context, and simultaneous engagement of GLP-1R [13,25].

Tirzepatide is the first dual GIP/GLP-1 receptor agonist approved for the treatment of type 2 diabetes and chronic weight management in adults. Its clinical efficacy establishes the therapeutic value of dual-receptor agonism, although clinical outcomes alone cannot determine the relative contribution of GIPR activation, GLP-1R activation, or the specific pharmacological properties of the molecule [37,39].

### 3.2. Dual GIP/GLP-1 Receptor Agonists—Mechanism of Action

Tirzepatide is a long-acting peptide engineered from the GIP sequence to activate both GIPR and GLP-1R. It does not activate the two receptors with identical potency. Experimental studies characterize tirzepatide as an imbalanced dual agonist with greater engagement of GIPR than GLP-1R. At GIPR, its activity resembles that of native GIP, whereas at GLP-1R, it preferentially promotes cAMP generation over β-arrestin recruitment and induces less receptor internalization than native GLP-1. These signaling properties may prolong receptor availability and contribute to its metabolic effects, although their precise clinical relevance remains incompletely defined [13,25,26,40].

#### 3.2.1. Pancreatic and Glycemic Effects

Activation of both receptors enhances glucose-dependent insulin secretion through complementary signaling in pancreatic β-cells. GIPR and GLP-1R primarily couple to Gαs, stimulating adenylate cyclase and increasing intracellular cAMP, with subsequent activation of protein kinase A and Epac2. These pathways promote calcium-dependent insulin granule exocytosis and amplify insulin secretion when glucose concentrations are elevated. GLP-1R activation additionally contributes to the suppression of glucagon secretion through glucose-dependent and partly indirect mechanisms. Together, these effects improve postprandial glucose control while maintaining a relatively low intrinsic risk of hypoglycemia in the absence of concomitant insulin or insulin secretagogues [6,13,25,26,40].

#### 3.2.2. Regulation of Appetite and Central Nervous System Pathways

Reduced energy intake is a major determinant of the weight loss achieved with tirzepatide. GLP-1R activation engages vagal, brainstem, and hypothalamic pathways regulating satiation and satiety. Preclinical studies suggest that central GIPR signaling may additionally modulate neuronal circuits involved in food intake, nutrient preference, and reward processing. However, direct evidence demonstrating that GIPR activation enhances appetite suppression in humans remains limited. The greater weight reduction observed with tirzepatide therefore cannot yet be attributed conclusively to a specific central synergy between GIPR and GLP-1R [14,25,26,40].

#### 3.2.3. Effects on Adipose Tissue and Metabolic Function

GIPR is expressed in adipose tissue, where physiological GIP signaling influences lipid uptake, adipocyte function, and postprandial nutrient storage. Under pharmacological conditions, sustained GIPR activation combined with GLP-1R agonism may improve adipose tissue insulin sensitivity, lipid handling, and metabolic flexibility. More effective storage of lipids within metabolically functional adipose tissue could theoretically limit ectopic lipid deposition in the liver and skeletal muscle. Nevertheless, these mechanisms are supported mainly by experimental evidence, and some of the improvements observed during tirzepatide treatment may be secondary to weight loss and improved glycemic control rather than direct actions on adipocytes [7,13,25,26,40].

#### 3.2.4. Integrated Metabolic Effects

The efficacy of tirzepatide probably reflects the combined effects of reduced energy intake, enhanced glucose-dependent insulin secretion, improved insulin sensitivity, and favorable changes in lipid metabolism and nutrient partitioning. In adults with type 2 diabetes, tirzepatide produced greater reductions in glycated hemoglobin and body weight than semaglutide 1.0 mg in the SURPASS-2 trial [39]. More importantly for obesity treatment, the head-to-head SURMOUNT-5 trial demonstrated a mean body weight reduction of 20.2% with tirzepatide and 13.7% with semaglutide 2.4 mg after 72 weeks in adults with obesity without diabetes [41]. These findings establish the superior efficacy of tirzepatide under the conditions studied but do not, by themselves, prove a specific molecular synergy between GIPR and GLP-1R.

Evidence in children and adolescents remains substantially more limited. Results from SURPASS-PEDS support the glycemic efficacy of tirzepatide in young people with type 2 diabetes, but these findings cannot be directly extrapolated to pediatric obesity. Until results from dedicated obesity trials become available, the long-term efficacy, developmental safety, and optimal clinical positioning of dual GIP/GLP-1 receptor agonists in pediatric patients remain uncertain [42]. 

**Figure 2:**
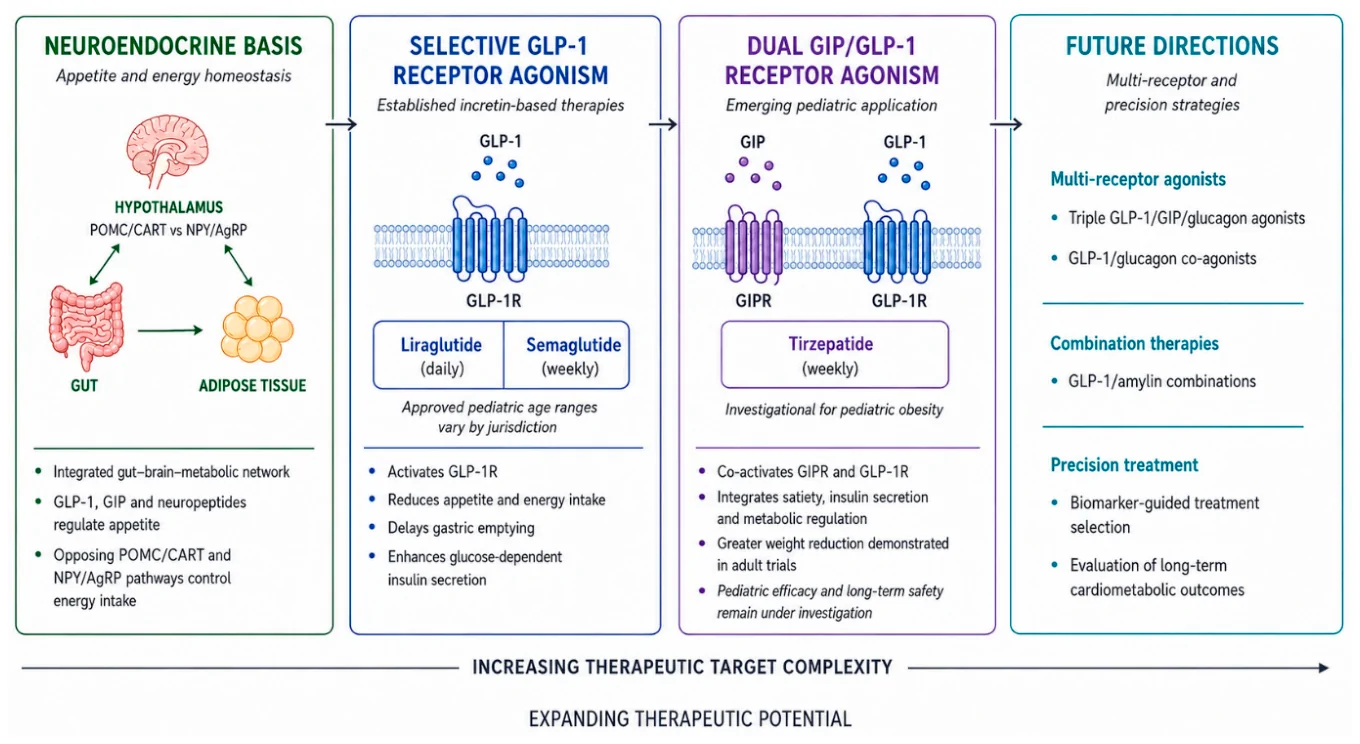
**From neuroendocrine mechanisms to clinical translation: evolution of incretin-based therapies in pediatric obesity.** This schematic summarizes how advances in neuroendocrine biology have been translated into successive generations of incretin-based therapies for pediatric obesity, from first-generation GLP-1 receptor agonists to emerging multi-agonist approaches. It highlights the progression from fundamental understanding of gut–brain signaling and energy homeostasis to clinically implemented GLP-1 receptor agonists, dual GIP/GLP-1 receptor agonists, and future therapeutic strategies. The horizontal arrows represent the increase in biological complexity across successive generations of incretin-based interventions. Approved pediatric obesity indications for liraglutide and semaglutide vary by agent and jurisdiction, whereas tirzepatide remains investigational for pediatric obesity. Abbreviations: GIP, glucose-dependent insulinotropic polypeptide; GLP-1, glucagon-like peptide-1; GLP-1R, glucagon-like peptide-1 receptor; GIPR, glucose-dependent insulinotropic polypeptide receptor; POMC, proopiomelanocortin; CART, cocaine- and amphetamine-regulated transcript; NPY, neuropeptide Y; AgRP, agouti-related peptide.

The transition from the neuroendocrine mechanisms of incretin action to their clinical applications in paediatric obesity is illustrated in Figure 2.

## 4. Clinical Evidence for Incretin-Based Therapies in Children and Adolescents

### 4.1. Liraglutide

Liraglutide was the first GLP-1 receptor agonist approved for chronic weight management in adolescents with obesity. Its efficacy was established in a randomized, double-blind, placebo-controlled trial involving 251 adolescents aged 12 to <18 years who received liraglutide 3.0 mg once daily or placebo, both combined with lifestyle intervention, for 56 weeks. The estimated between-group difference in the change in BMI standard deviation score was −0.22 in favor of liraglutide. A reduction in BMI of at least 5% was achieved by 43.3% of participants receiving liraglutide and 18.7% receiving placebo, whereas reductions of at least 10% occurred in 26.1% and 8.1%, respectively [43,44,45].

The magnitude of weight reduction was moderate compared with that subsequently reported for semaglutide, but the trial established the clinical efficacy of GLP-1 receptor agonism in adolescent obesity. Gastrointestinal symptoms, particularly nausea, vomiting, and diarrhea, were the most frequently reported adverse events. Partial loss of the treatment effect after discontinuation also indicated that continued therapy may be required to maintain weight-related benefits [43].

More recent evidence has extended the evaluation of liraglutide to younger children. In the SCALE Kids trial, 82 children aged 6 to <12 years with obesity were randomly assigned to liraglutide or placebo for 56 weeks, in addition to lifestyle intervention. The mean change in BMI was −5.8% with liraglutide and +1.6% with placebo, corresponding to an estimated treatment difference of −7.4 percentage points. A BMI reduction of at least 5% was achieved by 46% of children receiving liraglutide compared with 9% receiving placebo [46].

These findings supported extension of the European indication to children aged 6 to <12 years with obesity and a body weight of at least 45 kg. In the United States, however, liraglutide remains approved for chronic weight management in adolescents aged ≥12 years with a body weight above 60 kg. Regulatory indications should therefore be presented separately according to jurisdiction [44,45,46].

### 4.2. Semaglutide

Semaglutide is a long-acting GLP-1 receptor agonist administered once weekly. Its prolonged half-life and sustained receptor engagement permit less frequent administration than liraglutide. Although semaglutide has produced greater mean weight reduction in pediatric trials, no direct head-to-head trial comparing semaglutide with liraglutide has been conducted in adolescents. Consequently, differences in efficacy should not be attributed solely to receptor affinity or central nervous system activity [44,45,47].

The pivotal STEP TEENS trial included 201 adolescents aged 12 to <18 years with obesity or overweight and at least one weight-related comorbidity. Participants received once-weekly semaglutide 2.4 mg or placebo for 68 weeks, together with lifestyle intervention. The mean percentage change in BMI was −16.1% with semaglutide and +0.6% with placebo, corresponding to a treatment difference of −16.7 percentage points. At least 5% body weight reduction was achieved by 73% of participants receiving semaglutide compared with 18% receiving placebo [47].

Semaglutide also produced improvements in waist circumference, glycated hemoglobin, several lipid parameters, and alanine aminotransferase concentrations. Gastrointestinal adverse events occurred more frequently with semaglutide than with placebo, and cholelithiasis was reported in 4% of participants receiving semaglutide. Nevertheless, the overall safety profile was broadly consistent with that previously observed in adults [47].

Among GLP-1 receptor agonists currently approved for pediatric obesity, semaglutide has produced the largest mean BMI reduction reported in a pivotal randomized trial. However, available evidence is limited primarily to a single 68-week study. Data regarding treatment beyond adolescence, long-term maintenance of weight reduction, effects on growth and pubertal development, and outcomes following discontinuation remain limited [3,47].

### 4.3. Other GLP-1 Receptor Agonists

Other GLP-1 receptor agonists, particularly exenatide, have also been evaluated in randomized studies involving children and adolescents with obesity. These studies generally demonstrated modest reductions in BMI and improvements in selected metabolic outcomes but were limited by small sample sizes, short treatment duration, and heterogeneous study populations. Neither exenatide nor other GLP-1 receptor agonists evaluated in these earlier trials are currently approved specifically for pediatric obesity. Nevertheless, their inclusion is important for presenting the complete development of incretin-based pharmacotherapy in this population [27,48,49,50,51].

### 4.4. Dual GIP/GLP-1 Receptor Agonists

Tirzepatide is the first dual GIP/GLP-1 receptor agonist approved for type 2 diabetes and chronic weight management in adults. Its greater efficacy compared with selective GLP-1 receptor agonism in adult obesity has generated considerable interest in its potential pediatric application. However, no randomized trial results evaluating tirzepatide specifically for obesity in children or adolescents had been published as of July 2026 [37,41,44,45].

Two phase III trials are evaluating tirzepatide in adolescents with obesity. SURMOUNT-ADOLESCENTS (NCT06075667) includes adolescents with obesity or overweight and at least one weight-related comorbidity. The study is active but no longer recruiting, and its results were not publicly available as of July 2026. SURMOUNT-ADOLESCENTS-2 (NCT06439277) is recruiting adolescents with obesity and multiple weight-related comorbidities and includes an extended treatment and follow-up period [52].

Indirect pediatric evidence is available from SURPASS-PEDS, a randomized phase III trial involving 99 participants aged 10 to <18 years with inadequately controlled type 2 diabetes. Tirzepatide produced substantial reductions in glycated hemoglobin and body mass index; at 30 weeks, the 10 mg dose was associated with an 11.2% reduction in BMI from baseline. Gastrointestinal symptoms were the most frequently reported adverse events, and the overall safety profile was consistent with that observed in adults [42].

These findings demonstrate that tirzepatide is pharmacologically active in pediatric patients, but they cannot be directly extrapolated to children and adolescents treated for obesity without diabetes. Differences in metabolic phenotype, treatment goals, baseline glycemic status, and risk–benefit considerations require evidence from dedicated obesity trials. Until such results become available, liraglutide and semaglutide remain the only incretin-based agents approved specifically for chronic weight management in pediatric populations, with indications varying according to age and regulatory jurisdiction [42,44,45].

### 4.5. Evidence from Meta-Analyses

Available meta-analyses have primarily included studies evaluating liraglutide and semaglutide; however, due to the lack of published results from randomized pediatric trials of tirzepatide, dual GIP/GLP-1 receptor agonists have not yet been incorporated into quantitative efficacy analyses. In recent years, several meta-analyses and systematic reviews have summarized the efficacy of GLP-1 receptor agonists in children and adolescents with obesity. The findings of these analyses are consistent and indicate that GLP-1 RA treatment results in a significantly greater reduction in BMI compared with placebo, as well as improvements in several metabolic parameters, including insulin resistance, fasting glucose levels, and lipid profile [48,49,50,51].

Recent meta-analyses provide a broader assessment of GLP-1 receptor agonist efficacy than individual pediatric trials. Sedenho-Prado et al. included eight randomized studies comprising 715 children and adolescents with obesity and demonstrated significant reductions in BMI, body weight, and waist circumference with GLP-1 receptor agonists compared with control treatment. The pooled analysis also showed a modest reduction in systolic blood pressure, whereas significant effects on lipid parameters, HbA1c, and fasting glucose were not consistently observed [53].

A more recent meta-analysis by Chen et al., comprising 14 randomized controlled trials and 1349 participants, confirmed significant reductions in body weight and BMI and additionally demonstrated modest improvements in HbA1c and fasting glucose. Importantly, this analysis included several GLP-1 receptor agonists—liraglutide, exenatide, semaglutide, and dulaglutide—indicating that the observed effect is not restricted to the two agents currently most commonly used for pediatric obesity [54].

Comparative evidence is provided by the network meta-analysis of Liu et al., which included 11 randomized trials with 953 participants and directly and indirectly compared semaglutide, liraglutide, exenatide, and dulaglutide. Semaglutide was associated with the greatest reductions in body weight, BMI, and BMI z-score and ranked as the most effective of the evaluated GLP-1 receptor agonists for anthropometric outcomes [49]. However, these findings should be interpreted cautiously because the differences between agents were based predominantly on indirect comparisons across trials differing in treatment duration, dose, study population, and concomitant lifestyle intervention rather than on head-to-head randomized comparisons.

Across pooled analyses, gastrointestinal adverse events, particularly nausea and vomiting, were more frequent with GLP-1 receptor agonists than with placebo, although treatment discontinuation was uncommon. Available meta-analyses remain insufficiently powered to assess uncommon adverse events or long-term safety outcomes, particularly those related to growth, pubertal development, gallbladder disease, pancreatitis, and the consequences of prolonged exposure beginning in childhood [48,49,50,51,53,54].

Notably, current pediatric meta-analyses primarily evaluate selective GLP-1 receptor agonists. Published randomized obesity trials of tirzepatide in children and adolescents were not available as of July 2026; therefore, dual GIP/GLP-1 receptor agonism has not yet been incorporated into quantitative evidence syntheses specifically addressing pediatric obesity.

### 4.6. Body Composition, Bone Accrual, Growth and Puberty: A Critical Evidence Gap

Changes in BMI alone provide limited information on the composition and developmental consequences of weight loss in adolescents. Adult data suggest that approximately 25–33% of weight lost during treatment with GLP-1 receptor agonists or tirzepatide may be derived from lean mass. In the SURMOUNT-1 trial, approximately 75% of weight loss was attributable to fat mass and 25% to lean mass [55]. Whether a similar pattern occurs in adolescents remains uncertain. Although pediatric trials of liraglutide and semaglutide have demonstrated clinically meaningful reductions in BMI, comprehensive assessment of body composition using dual-energy X-ray absorptiometry (DXA) (GE HealthCare, Chicago, Illinois, United States)has not been a primary endpoint in these studies [43,47]. Consequently, the extent to which pharmacological weight loss during adolescence affects adipose tissue relative to lean mass remains insufficiently characterized.

This question is particularly relevant during adolescence, a critical period of skeletal growth and bone mineral accrual. Bone development is influenced by linear growth, pubertal maturation, body composition, physical activity, and nutritional status [56,57]. Although weight reduction may improve insulin sensitivity and reduce obesity-related metabolic and inflammatory abnormalities, prolonged negative energy balance and reduced mechanical loading could potentially limit lean tissue accretion and bone formation [57,58]. Moreover, the effects of sustained GIP receptor agonism on the developing skeleton remain unknown. Thus, the overall impact of incretin-based pharmacotherapy on bone accrual during adolescence cannot currently be established.

Potential effects on linear growth and pubertal development also warrant consideration. Energy availability interacts with the GH–IGF-1 and hypothalamic–pituitary–gonadal axes, while changes in adipose tissue may influence leptin signaling and the kisspeptin–GnRH pathway. However, current evidence is insufficient to determine whether the magnitude or rate of weight loss achieved with GLP-1/GIP receptor agonists can meaningfully affect growth velocity, pubertal tempo, menstrual regularity, or ultimately attained adult height. Importantly, existing pediatric trials were neither designed nor powered to evaluate these outcomes across the full course of growth and maturation [43,47,59].

Interpretation is further complicated by the effects of obesity itself on growth and pubertal development. Childhood obesity may be associated with accelerated linear growth, advanced bone age, and earlier pubertal development, particularly in girls [59,60]. Therefore, a reduction in growth velocity or a change in pubertal progression during treatment should not automatically be interpreted as treatment-related developmental suppression; in some cases, it may instead reflect a shift toward a more physiologically appropriate developmental trajectory. Distinguishing between these possibilities requires longitudinal assessment of growth velocity, bone age, pubertal staging, body composition, and, ultimately, attained adult height.

Until such evidence becomes available, pediatric treatment with GLP-1-based therapies should be accompanied by attention to nutritional adequacy, particularly with respect to protein, calcium, and vitamin D intake, together with regular physical activity, including resistance exercise. Future clinical trials should incorporate standardized longitudinal assessments of body composition, bone health, growth, and pubertal development to determine whether the metabolic benefits of pharmacological weight reduction can be achieved without compromising normal adolescent development.

## 5. Clinical Implementation of GLP-1 Receptor Agonists in Pediatric Obesity

### 5.1. Indications and Patient Selection

Pediatric obesity is a chronic, relapsing disease that may require the early integration of pharmacotherapy into comprehensive treatment. The American Academy of Pediatrics recommends offering weight-management pharmacotherapy to adolescents aged ≥12 years with obesity, according to medication-specific indications, risks, and benefits, as an adjunct to intensive health behavior and lifestyle treatment. Importantly, initiation of pharmacotherapy does not require documented failure of lifestyle intervention. Instead, treatment intensity should be matched to obesity severity, the presence of complications, and the individual needs of the patient [1,2,3,51].

Regulatory indications vary by jurisdiction and should therefore be stated precisely. In the European Union, liraglutide is approved for adolescents aged ≥12 years with obesity and a body weight >60 kg and, following an extension of its indication based on the SCALE Kids trial, children aged 6 to <12 years with obesity and a body weight ≥45 kg. In the United States, liraglutide is currently approved for adolescents aged ≥12 years with obesity and a body weight >60 kg. Semaglutide is approved in both the European Union and the United States for weight management in adolescents aged ≥12 years with obesity; the European indication additionally requires a body weight >60 kg [44,45].

Patient selection should account for age and local regulatory indications, obesity severity, developmental stage, previous treatment history, obesity-related complications, contraindications, concomitant medications, and the preferences and readiness of the patient and family. The presence of prediabetes or type 2 diabetes mellitus, hypertension, dyslipidemia, metabolic dysfunction-associated steatotic liver disease (MASLD), obstructive sleep apnea, polycystic ovary syndrome, orthopedic complications, or clinically significant psychosocial impairment may increase the urgency of pharmacological intervention. However, pharmacotherapy should not be restricted exclusively to patients who have already developed such complications when medication-specific obesity criteria are otherwise fulfilled [1,2,3,51].

As of 2026, tirzepatide is not approved for the treatment of pediatric obesity. Although it has received authorization for the treatment of type 2 diabetes mellitus in pediatric patients aged ≥10 years in the United States and the European Union, its use specifically for obesity in children and adolescents remains investigational [44,45].

### 5.2. Positioning of Pharmacotherapy Within Comprehensive Obesity Care

Pharmacotherapy should be integrated with, rather than sequentially added after, intensive health behavior and lifestyle treatment. Comprehensive care should include age-appropriate nutritional support, physical activity, behavioral strategies, family involvement, and psychological support when indicated. The purpose of these interventions is not only to enhance weight reduction but also to support nutritional adequacy, physical fitness, psychological well-being, and the long-term sustainability of treatment outcomes [1,2,3].

Treatment goals should extend beyond changes in BMI and include improvement in metabolic health, obesity-related complications, physical functioning, quality of life, and control of hunger and eating-related distress. GLP-1 receptor agonists may facilitate adherence to nutritional interventions by reducing hunger, increasing satiety, and decreasing energy intake; nevertheless, they do not replace the broader components of multidisciplinary obesity care [1,2,3,51].

The decision to initiate treatment should be based on shared decision-making involving the patient, caregivers, and multidisciplinary clinical team. The expected benefits should be balanced against possible adverse events, injection burden, treatment accessibility, costs, family preferences, and the likelihood that long-term therapy will be feasible. Patients and caregivers should be informed that obesity is a chronic disease and that pharmacotherapy may require prolonged administration to maintain its effects [1,2,3,51].

### 5.3. Initiation, Monitoring, and Long-Term Management

Before treatment initiation, clinical assessment should include anthropometric measurements, blood pressure, growth velocity, pubertal development, obesity-related complications, concomitant medications, and potential contraindications. Laboratory evaluation should be individualized according to age and clinical presentation and may include assessment of glucose metabolism, lipid profile, and liver function. Mental health, disordered eating, and the possible presence of an eating disorder should also be evaluated before and during therapy [1,2,3,44,45,47].

GLP-1 receptor agonists should be initiated and titrated according to the product-specific prescribing information. Gradual dose escalation is important for reducing gastrointestinal adverse events. Patients and caregivers should receive counseling regarding nausea, vomiting, diarrhea, constipation, hydration, and symptoms that may indicate gallbladder disease or pancreatitis. The risk of hypoglycemia is generally low when GLP-1 receptor agonists are used without insulin or insulin secretagogues but should be considered in patients receiving these medications for concomitant diabetes [44,45,47].

Follow-up should include assessment of treatment efficacy, tolerability, adherence, nutritional intake, and psychosocial well-being. Therapeutic response should be evaluated using age-appropriate measures, including BMI trajectory, BMI z-score or percentage of the 95th BMI percentile, as well as changes in obesity-related complications and quality of life. Particular attention should be given to growth velocity and pubertal development because the long-term developmental effects of incretin-based therapy remain incompletely characterized [1,2,3,44,45,47].

**Figure 3:**
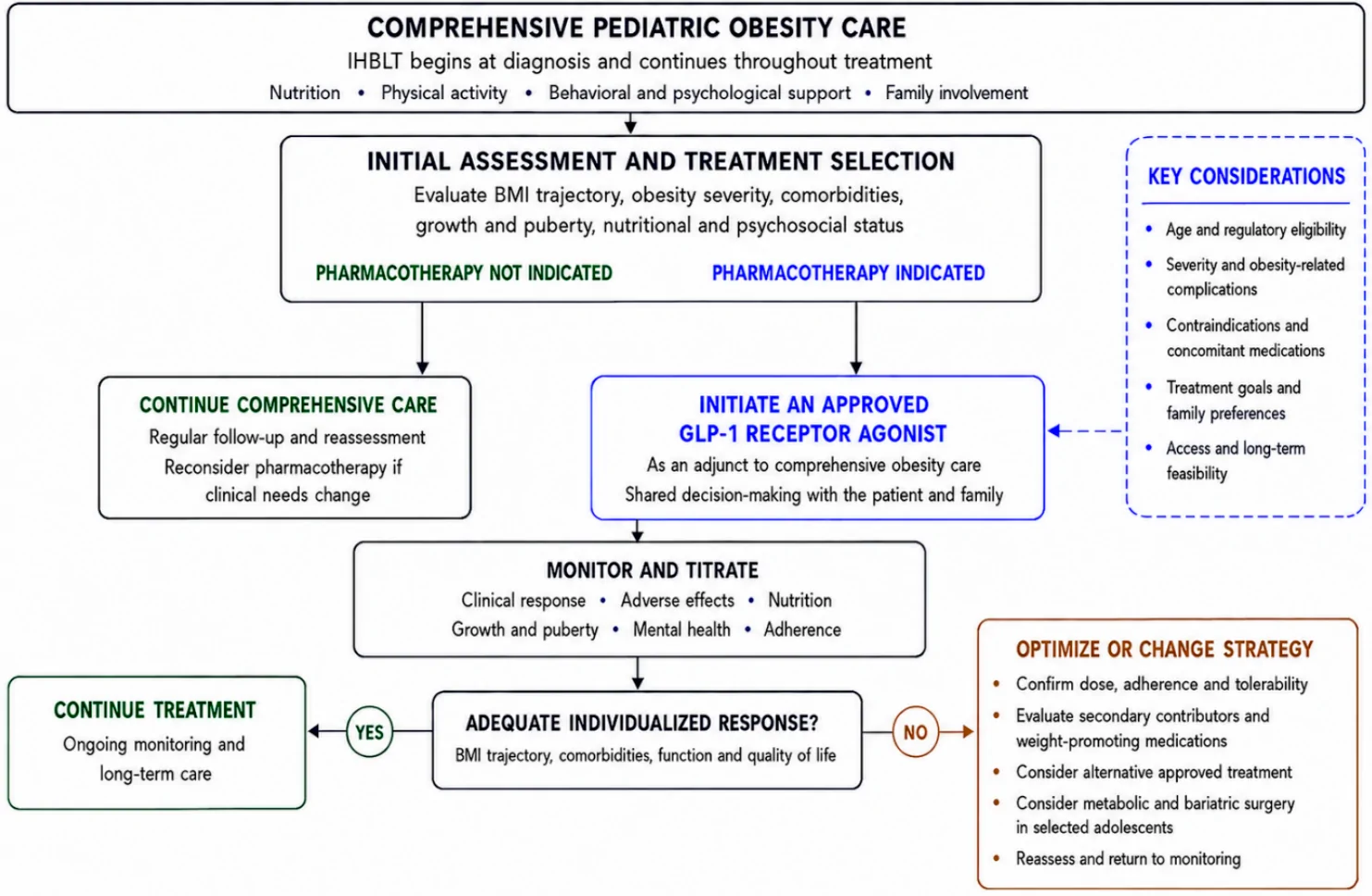
**Clinical algorithm for the use of GLP-1 receptor agonists within comprehensive pediatric obesity care.** This algorithm summarizes a stepwise approach to the management of pediatric obesity, integrating lifestyle intervention as the foundation of treatment with evidence-based use of incretin therapy in selected patients. Eligibility for pharmacotherapy is assessed at the initial evaluation and reconsidered during follow-up; medication is used as an adjunct to comprehensive obesity care when indicated. Ongoing management includes regular monitoring of treatment efficacy, safety and tolerability, growth and pubertal development, adherence, and psychosocial well-being. In patients with inadequate response, further therapeutic optimization and, in selected adolescents with severe obesity, consideration of metabolic and bariatric surgery may be appropriate following comprehensive multidisciplinary evaluation. Abbreviations: BMI, body mass index; GLP-1, glucagon-like peptide-1; IHBLT, intensive health behavior and lifestyle treatment.

Continuation or discontinuation criteria should follow the relevant product information and local guidelines, as no single response threshold applies universally to all GLP-1 receptor agonists. In patients with an inadequate response, clinicians should reassess adherence, dose escalation, tolerability, concomitant medications, secondary causes of weight gain, and the need for alternative or additional treatment strategies, this is illustrated in Figure 3. These may include another anti-obesity medication or, in appropriately selected adolescents with severe obesity, evaluation for metabolic and bariatric surgery [1,44,45,47].

Discontinuation should be planned carefully because weight regain commonly occurs after withdrawal of incretin-based therapy. Continued nutritional, behavioral, and clinical support is therefore essential, regardless of whether pharmacotherapy is maintained, changed, or discontinued [2,43].

### 5.4. Tolerability, Adherence and Treatment Discontinuation in Real-World Pediatric Practice

Adverse-event profiles reported in registration trials may not fully reflect the treatment burden associated with tolerability in routine pediatric practice. Trial participants are subject to close monitoring, structured dose escalation, and regular support, whereas these conditions may not always be available in routine care. Real-world data in adults indicate that a substantial proportion of patients discontinue incretin-based therapies within 12 months, with gastrointestinal symptoms, treatment costs, and loss of perceived benefit among commonly reported reasons. Pediatric real-world data remain limited, although emerging observational evidence suggests that treatment discontinuation may also be substantial in this population [47,61].

In the pediatric population, weekly subcutaneous injections, needle-related anxiety, and persistent nausea and vomiting may be particularly relevant and may negatively affect school attendance, physical activity, and social functioning. These symptoms may lead to missed doses without informing healthcare providers. Chronic nausea and reduced food intake may also have nutritional consequences during a period of rapid growth, particularly regarding the intake of protein, calcium, iron, and other micronutrients. This further highlights the importance of adequate nutritional monitoring and assessment of body composition during pharmacological weight management [62].

Adverse events should also be interpreted in absolute rather than solely relative terms. Cholelithiasis occurred in approximately 4% of adolescents treated with semaglutide in the STEP TEENS trial, compared with none in the placebo group, representing an important safety signal in this age group. Some cases of acute gallbladder disease required intervention. Rapid or substantial weight loss is a recognized risk factor for gallstone formation, although the increased incidence of gallbladder disease may not be explained solely by the degree of weight loss [47]. Although pancreatitis is rare, it remains a clinically relevant potential adverse event that should be considered when discussing the risks and benefits of treatment with patients and their families [45].

In clinical practice, treatment effectiveness depends not only on the pharmacological efficacy of the drug but also on adherence and the ability to maintain therapy over the long term. Future pediatric studies should therefore systematically assess adherence, the frequency and reasons for treatment discontinuation, and effectiveness under conditions that more closely reflect routine clinical practice. Long-term pediatric registries should also be established to evaluate the tolerability, safety, treatment persistence, and real-world effectiveness of incretin-based therapies.

### 5.5. Ethical and Economic Implications of Potentially Indefinite Therapy

Weight regain after treatment discontinuation has been observed with incretin-based therapies and reflects their effects on the mechanisms regulating energy balance rather than treatment “failure.” Partial loss of treatment effects has been observed after liraglutide discontinuation in adolescents, while a loss of some of the treatment benefit was observed after treatment withdrawal in the STEP TEENS trial [47,61]. In adults, a substantial proportion of lost weight may be regained within one year after treatment discontinuation; in the STEP 1 trial extension, participants regained approximately two-thirds of their prior weight loss within one year after semaglutide withdrawal [62]. Thus, treatment initiated during the pediatric period may, based on current evidence, require long-term and potentially indefinite continuation, an issue that should be considered when making treatment decisions.

High treatment costs may limit access to therapy, particularly in the absence of full reimbursement, and may lead to treatment interruptions related to changes in the family’s financial circumstances [45].

## 6. Future Perspectives for Incretin-Based Therapies in Pediatric Obesity

Despite the short-term efficacy demonstrated in current clinical trials, major questions remain regarding the long-term use of GLP-1 receptor agonists and dual GIP/GLP-1 receptor agonists in children and adolescents. Of particular importance are their potential effects on linear growth, pubertal development, bone health, body composition, nutritional status, and neurodevelopment. Long-term studies are also needed to determine whether the metabolic benefits observed during treatment translate into sustained reductions in obesity-related morbidity and whether these benefits can be maintained after pharmacotherapy is discontinued [2,3,43,47,48,50].

Dual GIP/GLP-1 receptor agonism has not been evaluated in any randomized trial of pediatric obesity without diabetes, and tirzepatide is not approved for this indication in any jurisdiction. Studies conducted in adults indicate that simultaneous activation of both receptors may produce greater weight reduction and more favorable effects on glucose and lipid metabolism than selective GLP-1 receptor agonism. However, experience with this drug class in the pediatric population remains limited. Dedicated randomized trials are therefore required to establish its efficacy, developmental safety, optimal dosing, and long-term risk–benefit profile in children and adolescents with obesity [7,13,37,41,42].

The developmental questions identified throughout this review—the tissue composition of pharmacologically induced weight loss, bone mineral accrual during the pubertal window, height velocity and pubertal tempo, nutritional adequacy during sustained appetite suppression, and the development of eating behavior and interoceptive regulation—share a common structure: each is mechanistically plausible, none has been measured in a pediatric trial, and each requires follow-up extending beyond the 56- to 68-week horizon of the existing evidence. Prioritizing these outcomes in the design of ongoing and future pediatric trials is, in our view, more consequential for this population than further characterization of short-term anthropometric efficacy.

An important and largely unexplored area is the effect of GLP-1 and dual GIP/GLP-1 receptor agonists on the developing respiratory system. Pediatric obesity is associated with impaired respiratory mechanics, obstructive sleep apnea, and a more severe course of childhood asthma; consequently, treatment-induced weight reduction may indirectly improve respiratory outcomes. Preclinical studies and emerging evidence from adults additionally suggest that GLP-1 receptor signaling may modulate airway inflammation and immune responses, although direct respiratory effects independent of weight loss have not been established in pediatric populations. Future studies should therefore incorporate respiratory outcomes, including pulmonary function, asthma control, sleep-disordered breathing, and exercise tolerance, while also evaluating the long-term safety of these therapies in the developing respiratory system [1,63,64].

Another major challenge is the substantial interindividual variability in therapeutic response. Future research should aim to identify clinical, metabolic, genetic, behavioral, and developmental predictors of treatment efficacy and tolerability. Such markers could facilitate selection of the most appropriate medication, support individualized dose escalation, and identify patients who may require alternative or combined treatment strategies. Particular attention should also be given to the preservation of lean mass and bone health, nutritional adequacy during rapid weight loss, and the psychological effects of long-term treatment.

Finally, the optimal duration of therapy remains uncertain. Because weight regain frequently occurs after treatment withdrawal, studies should evaluate maintenance strategies, including continued treatment at individualized doses, structured lifestyle support, sequential pharmacotherapy, and carefully planned discontinuation. As incretin-based therapies become increasingly available, their successful implementation will depend not only on efficacy but also on long-term safety, accessibility, treatment adherence, and integration into comprehensive, developmentally appropriate pediatric obesity care [2,43,47].

## 7. Conclusions

Incretin-based therapies translate advances in the understanding of gut–brain signaling into mechanism-based treatment of pediatric obesity. By targeting neuroendocrine pathways involved in appetite, satiety, gastric emptying, and glucose homeostasis, GLP-1 receptor agonists can produce clinically meaningful reductions in BMI and improvements in selected cardiometabolic risk markers when integrated into comprehensive obesity care. Among currently available agents, semaglutide has produced the largest mean BMI reduction reported in a randomized trial involving adolescents, whereas liraglutide has provided evidence across a broader pediatric age range, including children aged 6 to <12 years.

However, current evidence is based predominantly on trials of limited duration and does not yet establish durable prevention of obesity-related morbidity or long-term developmental safety. Dual GIP/GLP-1 receptor agonism is mechanistically plausible but has not been evaluated in pediatric obesity, and its role must be defined by dedicated clinical trials. Incretin-based therapies should therefore be integrated with nutritional, behavioral, psychological, and family-based interventions, with individualized patient selection and careful monitoring of growth, pubertal development, nutritional status, mental health, body composition, and adverse events. The central challenge is now to determine whether the substantial short-term efficacy of these therapies can be translated into durable, accessible, and developmentally safe benefits throughout childhood and adolescence.

## Data Availability

No new data were created or analyzed in this study. Data sharing is not applicable to this article.
